# Supporting the implementation of new healthcare technologies by investigating generalisability of pilot studies using area-level statistics

**DOI:** 10.1186/s12913-022-08735-3

**Published:** 2022-11-24

**Authors:** James Alexander Doorbar, Christopher S. Mathews, Karin Denton, Matejka Rebolj, Adam R. Brentnall

**Affiliations:** 1 Cancer Prevention Group, School of Cancer & Pharmaceutical Sciences, Faculty of Life Sciences & Medicine, King’s College London, London, UK; 2 Severn Pathology, Southmead Hospital, North Bristol NHS Trust, Bristol, UK; 3 Wolfson Institute of Population Health, Queen Mary University of London, London, UK

**Keywords:** Generalisability, Pilot studies, Population statistics, Screening, Process indicators, Human papillomavirus testing, Technology implementation

## Abstract

**Background:**

Implementation of new technologies into national health care systems requires careful capacity planning. This is sometimes informed by data from pilot studies that implement the technology on a small scale in selected areas. A critical consideration when using implementation pilot studies for capacity planning in the wider system is generalisability. We studied the feasibility of using publicly available national statistics to determine the degree to which results from a pilot might generalise for non-pilot areas, using the English human papillomavirus (HPV) cervical screening pilot as an exemplar.

**Methods:**

From a publicly available source on population indicators in England (“Public Health Profiles”), we selected seven area-level indicators associated with cervical cancer incidence, to produce a framework for post-hoc pilot generalisability analysis. We supplemented these data by those from publicly available English Office for National Statistics modules. We compared pilot to non-pilot areas, and pilot regimens (pilot areas using the previous standard of care (cytology) vs. the new screening test (HPV)). For typical process indicators that inform real-world capacity planning in cancer screening, we used standardisation to re-weight the values directly observed in the pilot, to better reflect the wider population. A non-parametric quantile bootstrap was used to calculate 95% confidence intervals (CI) for differences in area-weighted means for indicators.

**Results:**

The range of area-level statistics in pilot areas covered most of the spectrum observed in the wider population. Pilot areas were on average more deprived than non-pilot areas (average index of multiple deprivation 24.8 vs. 21.3; difference: 3.4, 95% CI: 0.2–6.6). Participants in HPV pilot areas were less deprived than those in cytology pilot areas, matching area-level statistics. Differences in average values of the other six indicators were less pronounced. The observed screening process indicators showed minimal change after standardisation for deprivation.

**Conclusions:**

National statistical sources can be helpful in establishing the degree to which the types of areas outside pilot studies are represented, and the extent to which they match selected characteristics of the rest of the health care system ex-post. Our analysis lends support to extrapolation of process indicators from the HPV screening pilot across England.

**Supplementary Information:**

The online version contains supplementary material available at 10.1186/s12913-022-08735-3.

## Background

When research studies provide beneficial evidence to justify a new health care intervention, the implementation is sometimes first studied in a pilot embedded into routine health care [1–3]. Although usually undertaken within a limited number of health care units, pilots can help us better understand factors that are relevant for a successful translation of research findings into routine practice within a specific health care system. These factors include the acceptability of the new technology for the target population and their health care providers, or the real-life feasibility of clinical management pathways. Well-designed pilot studies can help configure relevant health care infrastructure and/or identify operational bottlenecks before a full roll-out. Furthermore, the collected data can guide decisions on resource allocation.

A successful example of such a study has been the recently completed English human papillomavirus (HPV) screening pilot, which explored the feasibility of replacing liquid-based cytology (LBC) with HPV testing within the English Cervical Screening Programme (CSP). The pilot was recommended on the back of robust evidence from randomised controlled trials undertaken in multiple countries including England showing that HPV testing is more highly sensitive to detect high-grade cervical intraepithelial neoplasia (CIN2 +), whose treatment prevents cervical cancer [4, 5]. The pilot, which started in 2013, included around 1.3 million women whose screening samples were processed in six CSP laboratories. Results demonstrated the practicability, acceptability, adherence to protocol, and confirmation of test and triage performance [3]. The lessons learnt through the pilot increased the confidence for a national implementation of HPV-based primary screening, which is now complete. The pilot informed the contracting and the organisation of the services within the national screening programme and helped update relevant clinical protocols. Notably, the data informed the (planned) extension of screening intervals in the devolved nations of the UK [6] and informed the choice of the screening and triage tests [7]. Furthermore, the data were used to help define the reduction in the number of screening laboratories within the entire programme, from almost 50 smaller to eight larger laboratories [8]. The decision balanced an expected ~ 85% decrease in the required number of LBC slides under HPV-based primary screening, which was directly observed in the six pilot sites, and the CSP’s requirement for a minimum laboratory workload of 35,000 LBC samples per year, required to maintain appropriate skills and quality.

The English HPV screening pilot used a non-randomised allocation of tests between women invited for screening. This was done to enhance clinical safety through uniform pathways for each colposcopy clinic, which were all linked to specific pilot laboratories. The inclusion of laboratory sites for participation in the pilot relied on self-selection through a bespoke application process. The laboratories that expressed their interest were those that could not only support data collection but could also withstand the introduced complexity in their daily operational processes by having to run two different testing protocols in parallel for several years, without jeopardising patient safety or having reassurance that the new protocol, which affected critical organisational aspects such as staffing, would be ultimately proposed for standard-of-care implementation. The observation that only 15% of screened women would require cytology triage (i.e., an 85% decrease in the workload compared to cytology-based screening) directly determined the required number of screening laboratories across the country after a full national roll-out of HPV-based screening. A sufficiently different observed absolute value of this proportion in the pilot may have led to a different configuration of the national screening laboratory network.

After the implementation, decisions like this often affect the availability and the quality of routinely delivered care for large numbers of individuals, and in cancer screening these numbers can quickly run into millions. Hence, when considering wider implementation of a new technology it will be important to assess generalisability affecting interpretation of data from pilot studies. This is particularly pertinent when pilot areas are selected for practical reasons e.g., their proximity to the study team, the ability of local health care providers to support research studies, or a better availability of the relevant health care services. [9, 10]

The dependency of a successful implementation of a new health care technology on the information collected within pilot studies underscores the importance of involving health care units that cover the spectrum of units and populations that are not involved in the pilot. Although the outcomes of implementation pilot studies are often reported in scientific literature, [11–16] the generalisability of the collected data is sometimes claimed simply by virtue of e.g., a pilot being embedded in a routine health care setting or its large size. However, these factors alone cannot guarantee that similar observations would be made in non-pilot health care units for the following two reasons.

First, the pilot population might not include the full diversity of the population. In this case there will be no direct data from the pilot from unrepresented groups. For example, a pilot undertaken in only affluent areas would not include people from deprived areas. The pilot population may then differ from the wider population in important aspects related to implementation such as their ability to access health care, and disease risk. More broadly, while a larger pilot size helps reduce uncertainty and adds to the training experience of the health care personnel, a pilot undertaken with a narrow sample of units (such as only in less deprived areas) would be unable to provide robust information for country-wide capacity planning and guidance for successful implementation.

Second, even if all major societal groups are represented in the pilot population, it is often expected that the pilot population will not match the wider population on key attributes. For example, if the pilot includes a range of socioeconomic deprivation groups, but the majority are from more affluent areas. In such a case, a naïve expectation that unadjusted process indicators from the pilot will be matched in the wider population may be flawed.

While a specific focus on generalisability appears to have rarely been considered in the pilot study literature, it has been considered in other contexts for a long time. An actuarial method to estimate the expected number of deaths in a reference population was first developed in the eighteenth century [17]. This may be used to tackle the problem of comparing mortality rates between populations with different age structures. More generally, this and other standardisation methods use assumptions to help transport results from one study setting to another [18]. To address the apparent lack of methodology in the application of such methods to pilot studies, we produced a post-hoc analytical framework which studied the relationship between clinical process outcomes observed in a real-world pilot study and the size of potential ecological differences between pilot and non-pilot areas by using direct standardisation methods. We assumed that the extent of generalisability to areas outside of the pilot can be partly studied indirectly, since patient behaviour and outcomes are often associated with patient characteristics such as age, socioeconomic factors, comorbidities, and health-related behavioural risk factors [19]. Partly, therefore, the degree to which pilot data can be considered as directly generalisable to non-pilot areas may be assessed by the extent to which pilot-area populations match key characteristics of the overall population covered by the health care system. We used the English HPV pilot as the real-world example to test the feasibility of our analytical approach. Pilot and non-pilot areas were compared using area-level data that are available without restrictions online, to reduce barriers and increase acceptability of the proposed approach.

## Methods

### The English HPV pilot

**Fig. 1:**
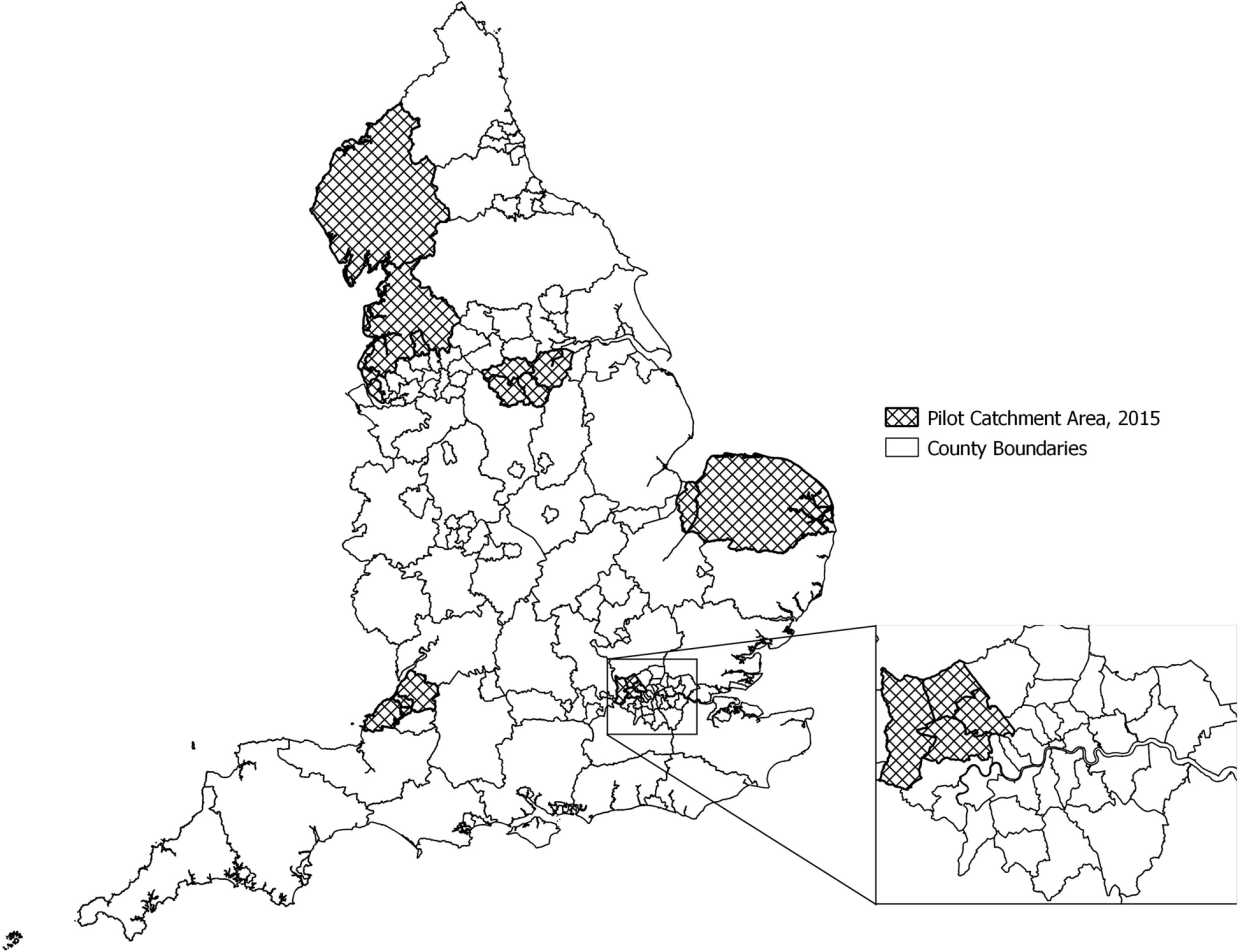
Catchment areas for pilot sites using the 2013–2015 definition. The figure was generated by authors using the QGIS software with information from the sources explained in the Methods

The English CSP invites women aged 25–49 every three years and women aged 50–64 every five years. The pilot used the same eligibility criteria [6]. The outcomes have been reported in detail previously [6, 7, 20–24]. The six pilot laboratories were distributed across England (Fig. 1). They previously participated in another large sentinel (pilot) study [25]. Each laboratory converted 20–40% of its screening workload to HPV testing. The two tests were allocated based on geographical area so that each provider could use a single clinical protocol. This mimicked the conditions under which a real-life implementation would take place and was expected to increase compliance with the protocols and reduce the risk of clinical errors. Screening tests were allocated in four sites (Liverpool, Manchester, Sheffield, and London’s Northwick Park) so that all general practices registered at an address belonging to a specific Clinical Commissioning Group (CCG) used the same test. CCGs are English geographical units for commissioning hospital and community NHS services at a local level [26]. In two sites (Bristol and Norwich), allocation was by general practice, and both tests could be used in a single CCG. The pilot’s first screening round took place between 2013 and 2016, a period during which various laboratory mergers were taking place. The largest differences in the catchment areas belonging to the pilot sites were seen between 2015 and 2016 (Supplementary Information Table S1 and Figure S1). Women continued to be followed up through the second screening round in three or five years, depending on their age.

### Data sources

#### General definitions

Overall, our analysis required three major groups of data:

1. Description of population characteristics. We aimed to compare the populations in the areas where the HPV pilot was taking place and the rest of England (non-pilot areas). Individual-level data describing the population’s characteristics are not routinely collected in England. Instead, a comprehensive set of health and related indicators aggregated to reflect the populations within specific administrative units is routinely published on a central publicly accessible portal. From the indicators included in that portal, we chose a selection of those that have been described in the literature as associated with either cervical cancer incidence or screening uptake. Each of these indicators is available for a specific type of an administrative unit. These differ between the indicators, and this informed the choice of administrative units for our analyses.
2. Organisation of the country into various types of administrative units. In England, each laboratory providing screening services to the CSP serves a defined catchment area, described by the boundaries of CCGs. Hence, the pilot area was defined in our study as the combined CCG areas served by the six laboratories that participated in the pilot (Fig. 1). The CCGs covering the rest of England were considered as non-pilot areas. Some of the cancer and screening indicators from the central portal included in our analysis were available by CCG. For indicators that were available for a different type of an administrative unit (usually county or unitary authority), we used information from a central geography portal to compare and translate the respective boundaries. By combining these two steps, we could determine whether the populations of the pilot areas differed from those in the non-pilot areas in terms of the selected indicators.
3. Individual-level clinical outcomes observed in the pilot. As the final step in our analysis, we determined the extent to which the clinical outcomes observed in the pilot areas were representative for the rest of England after considering the differences in the herein studied population characteristics. Individual-level data describing screening outcomes as observed in the pilot, which we had temporary access to under a contract to provide a comprehensive epidemiological analysis (see below in the section covering Declarations), were linked to the area-level characteristics as determined in the preceding steps, and up- or down-weighted as appropriate. We used the available information on individual women’s place of residence for linkage between the different data sets.

We explained these data items and how they were managed for inclusion in the analysis in detail below.

#### Area-specific cervical cancer incidence and screening uptake indicators

**Fig. 2:**
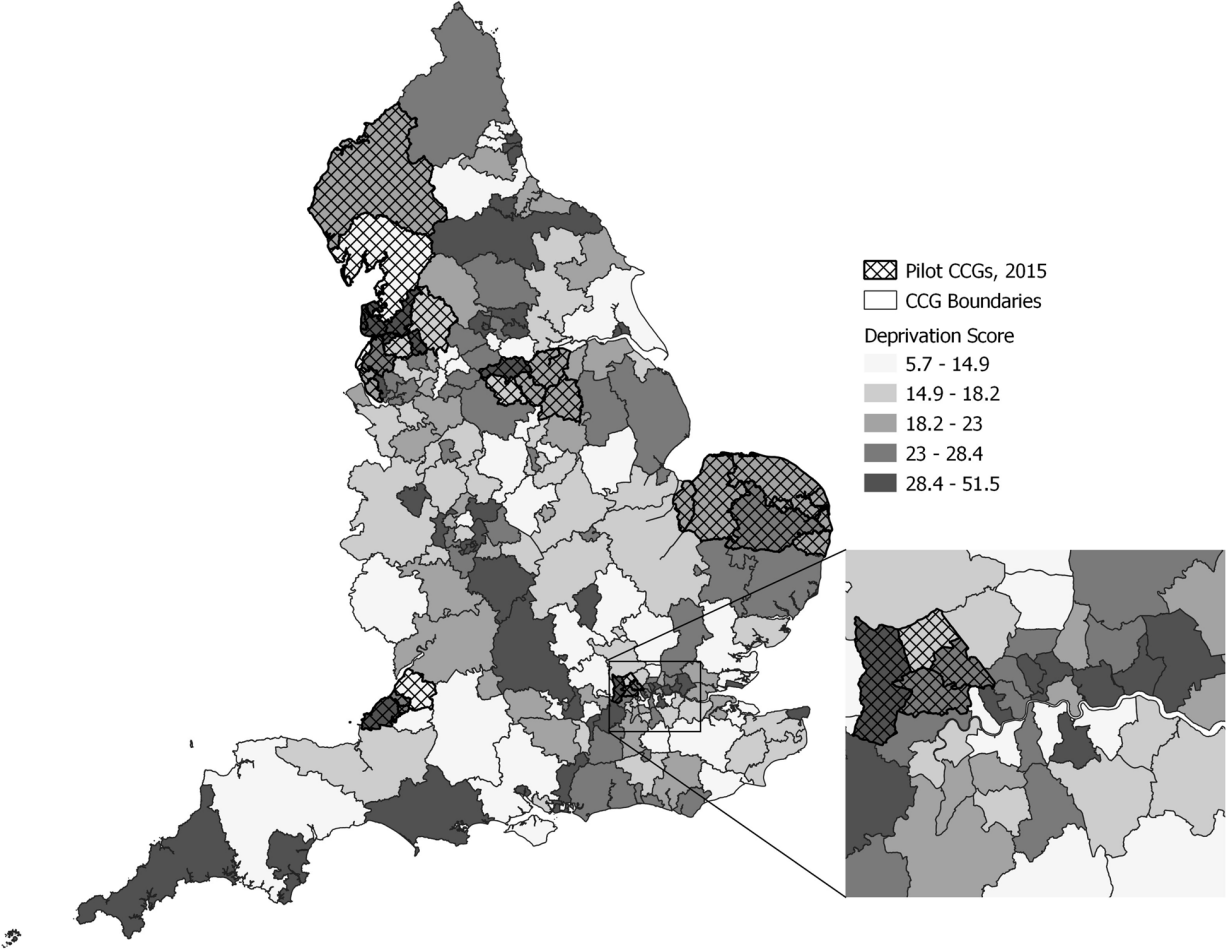
Distribution of IMD scores across England. Pilot CCGs were defined using the 2013–2015 definition. The figure was generated by authors using the QGIS software with information from the sources explained in the Methods

Population data by area for 2016 were obtained from the English Office for National Statistics (ONS) [27, 28]. The data on the population characteristics for the pilot and non-pilot areas were retrieved from the former Public Health England’s (now Office for Health Improvement and Disparities’) Public Health Profiles website (https://fingertips.phe.org.uk/). Detailed definitions for the selected seven ecological indicators are reported in Supplementary Information Table S2. These included 1) the incidence of cervical cancer diagnosis, which is lower where 2) screening coverage[29] and/or 3) HPV vaccination coverage[30, 31] are higher. Incidence is expected to increase with greater levels of 4) smoking prevalence [32], 5) socioeconomic deprivation [33], and 6) unprotected sexual contacts [34], the latter often indicated in a greater overall incidence of sexually transmitted infections (STI) [35]. Further, socioeconomic deprivation is associated with a lower screening coverage [36], but the coverage is higher in areas where 7) patients are more satisfied with their general practice (GP) surgeries [37]. Figure 2 shows the unequal distribution of socioeconomic deprivation scores across England. These are defined on the Index of Multiple Deprivation (IMD), a standard English area-based socioeconomic indicator [38]. The patterns for the remaining six indicators show similarly unequal distributions across the country (Supplementary Information Figure S2A-F). Values for areas with missing values or for areas that had disclosure controls applied due to small numbers were taken from previous years or were substituted with averages for the relevant geographic region.

#### Organisation of the country into administrative units

The laboratory sites generally had their catchment areas defined by CCG boundaries. We had available for analyses women’s postcodes associated with their first pilot sample, which were used to determine their residential CCGs as opposed to their GP’s postcode which linked to the pilot CCGs. In England, individuals can choose their GP within a specific distance from their home, so the women’s residential CCG usually overlaps with the GP’s CCG, with few exceptions. From the resulting list of women’s postcodes, we selected as pilot areas those CCGs where the number of samples registered in the pilot was at least 50% of the women who were screened [39]. CCGs were considered to have switched to HPV testing if over 80% of the baseline tests were reported with an HPV result, and to have continued using LBC if less than 20% of tests were reported with an HPV result. The remaining CCGs were defined as “mixed”. This was done separately for 2013–2015 and 2013–2016.

National statistics data on IMD, smoking, and screening coverage were available by CCG. Other indicators (population characteristics) were available by county/unitary authority (henceforth referred to as “county”). CCG boundaries from April 2017 and county boundaries from December 2017 were downloaded from the English ONS Open Geography Portal (a publicly available online resource) [40, 41]. Geographical overlaps between CCGs and counties were identified with spatial mapping software QGIS version 3.16.0 "Hannover". For most pilot CCGs, the boundaries coincided with those of the counties, with few exceptions. When part of a county was involved in the pilot (e.g., a single CCG), the county’s population was divided into pilot and non-pilot parts. CCG populations within a county were assigned that county’s indicator value, and the size of the population within the county was used for weighting. When a single CCG intersected with more than one county, the CCG population within each county was weighted separately and then analysed with respective county indicators.

#### Individual-level clinical outcomes observed in the pilot

Individual-level data generated during the pilot’s first (prevalence) screening round were retrieved from laboratory information systems [6, 7, 20–24]. We compared the age-specific values for screening process indicators directly observed in the pilot (with exact binomial 95% CI) and those obtained after standardisation by IMD quintile. The following process indicators having direct influence on the planning of capacities for triage testing, diagnostics and treatment were studied: the proportions of women with a positive screening test, a referral to colposcopy after baseline testing alone or in combination with two early recalls (at 12 and 24 months for HPV-positive women with a negative triage test at baseline), and with a diagnosis of high-grade cervical intraepithelial neoplasia (CIN2 + , CIN3 +).

### Statistical analysis

In the main analysis, we used the 2013–2015 definition for pilot areas (Supplementary Information Table S1). We first compared population characteristics using ecological indicators between the pilot and non-pilot areas, to gain insight into how representative and well matched the pilot population of eligible participants was for the whole country (external validity). We then compared ecological indicators between the populations of HPV and LBC pilot areas, to gain insight into how comparable the two pilot areas were for epidemiological analyses of the data (internal validity). In the latter analysis, the catchment areas of the two laboratories allocating screening tests by GP practice within a single CCG were excluded as the information on the allocation of tests by GP practice was not available for analysis; these involved 26% of the entire pilot population base. We did not report the analyses comparing pilot HPV and LBC areas for the four indicators where data were only available by county, as one large county included both HPV and LBC CCGs. Including this county among those with “mixed” testing would push the proportion of the pilot population base excluded from the analysis to 42%.

For each comparison, the mean values on the seven population indicators were obtained as a weighted average, with CCG- or county-specific weights defined as the proportion of women aged 25–64 years out of the national total. We calculated 95% confidence intervals (CI) for difference in means between the compared areas using a non-parametric quantile bootstrap with 5000 replicates.

For standardisation of absolute values of process indicators observed directly in the pilot, we used CCG-level (aggregate) IMD data, as individual-level values were not available for the non-pilot population. The populations of all 207 English CCGs were first ranked according to their CCG’s IMD score. From this ranked list, we derived IMD score quintiles, each of which contained approximately 20% of all female residents in England aged 25–64 years. The thus derived quintile category for each CCG was then added to the pilot datafile, using each woman’s residential CCG for linkage. Finally, screening process data for each woman was down- or upweighted according to her CCG-based IMD quintile so that the standardised HPV screening outcomes would match England.

Statistical analyses were undertaken using R version 3.6.3.

## Results

**Fig. 3:**
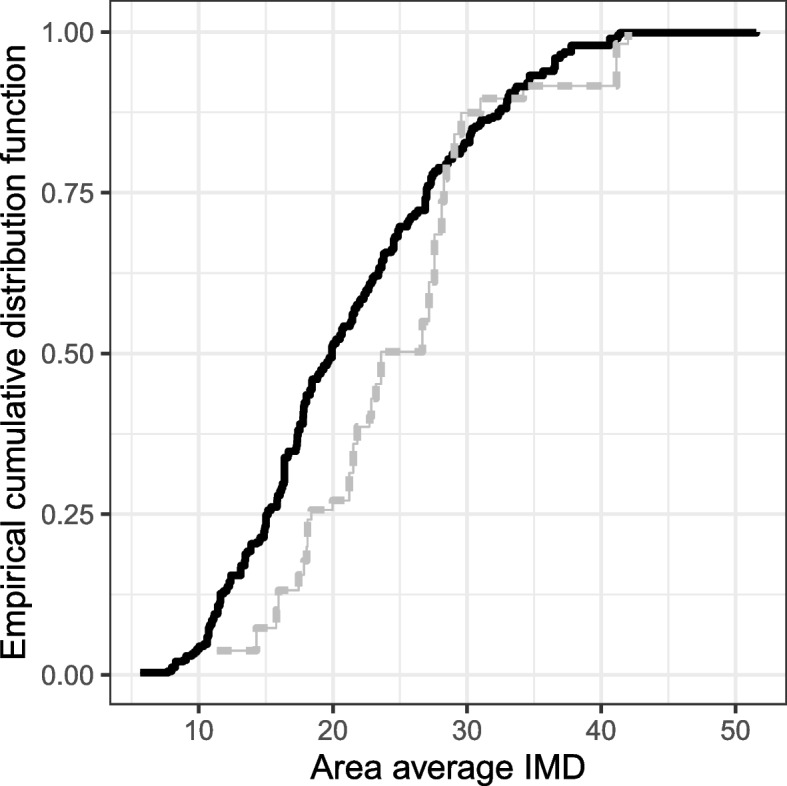
The estimated empirical cumulative distribution function for IMD scores comparing pilot and non-pilot areas. Pilot areas were defined using the 2013–2015 definition. Legend: Black line: non-pilot areas, Gray line: pilot areas

Pilot areas included a broad range of area-level statistics for IMD, that covered most of the range of the distribution seen in non-pilot areas. However, no pilot CCGs had an average aggregate IMD score of less than 11, compared with approximately 10% in the non-pilot areas (see cumulative distribution of IMD scores by CCG in Fig. 3). As a result, the average weighted IMD score was slightly higher in pilot areas (24.8) compared to non-pilot areas (21.3; difference of 3.4, 95% CI: 0.2–6.6; Table 1), confirming that the population in pilot areas was more deprived on average than the rest of England combined. In total, 1% of women undergoing screening with HPV testing in the pilot were from the least deprived IMD quintile measured at the aggregate CCG level, 23% from the second least deprived, 44% from the middle, 22% from the second most deprived, and 11% from the most deprived quintile. Despite this squashed and skewed distribution (the reference is 20% in each), standardisation by IMD quintile to better match the English population did not greatly change process indicators (Table 2). This was because the differences in process indicators between CCG quintiles were not large (Supplementary Information Table S3). The greatest relative differences between the standardised and observed values were found in older women aged 50–64 years, but few women in this age group have screen-detected abnormalities and require diagnostics and treatment.

**Table 1:** Comparison of population characteristics for pilot vs. non-pilot and pilot HPV vs. pilot LBC areas. Pilot areas were defined using the 2013–2015 definition. The comparison of HPV and LBC pilot areas was based on four laboratory sites

Indicator	Pilot (weighted mean, SD)	Non-pilot (weighted mean, SD)	Difference pilot vs. non-pilot (95% CI)	HPV testing (weighted mean, SD)	LBC (weighted mean, SD)	Difference HPV vs. LBC (95% CI)	National range (min–max per CCG)
IMD score	24.8 (7.3)	21.3 (8.1)	3.4 (0.2 to 6.6)	24.2 (6.3)	27.4 (7.3)	-3.2 (-9.1 to 3.2)	5.7–51.6
Smoking prevalence	19.1 (2.8)	18.3 (2.6)	0.8 (-0.3 to 1.9)	18.4 (2.3)	19.7 (3.1)	-1.3 (-3.3 to 1.3)	12.3–27.1
Cervical screening coverage	72.7 (4.3)	73.8 (3.9)	-1.2 (-3.1 to 0.6)	73.5 (3.4)	71.0 (4.9)	2.5 (-1.7 to 5.9)	56.8–83.1
HPV vaccination	83.9 (8.1)	83.7 (6.8)	0.2 (-4.6 to 4.0)				56.7–96.7
Incidence of STI per 100,000	784.1 (309.3)	801.6 (516.6)	-17.4 (-180.3 to 170.8)				365.0–3475.0
Incidence of cervical cancer per 100,000	10.6 (2.8)	9.8 (2.1)	0.9 (-0.6 to 2.1)				4.60–16.6
Satisfaction with GP surgery	72.7 (3.2)	73.4 (4.8)	-0.7 (-2.7 to 1.0)				58.6–96.7

Note: In this analysis, the pilot area included 29 CCGs and 18 counties (as relevant), and the non-pilot area included the remaining 178 CCGs and 134 counties and unitary authorities. In the analysis of the pilot HPV testing vs. pilot LBC areas, 21 CCGs and 13 counties and unitary authorities were included (the remaining pilot CCGs/counties belonged to the two excluded pilot laboratories that allocated both screening tests within the same CCGs)

*Abbreviations*. *GP* General practice/practitioner, *HPV* Human papillomavirus, *IMD* Index of multiple deprivation, *STI* Sexually transmitted infections

**Table 2:** Observed values for screening process indicators after HPV testing in the English pilot (first screening round in 2013–2016), and values after standardisation by IMD quintile

Process indicator	**24–29 years**			**30–49 years**			**50–64 years**		
	**N with outcome**	**Observed % (95% CI)**	**Standardised %**	**N with outcome**	**Observed % (95% CI)**	**Standardised %**	**N with outcome**	**Observed % (95% CI)**	**Standardised %**
N screened	76,277			224,400			103,206		
HPV positive	20,544	26.9% (26.6 to 27.2)	26.5%	22,563	10.1% (9.9 to 10.2)	9.9%	5486	5.3% (5.2 to 5.5)	5.7%
Referred at baseline	7898	10.4% (10.1 to 10.6)	10.8%	7049	3.1% (3.1 to 3.2)	3.2%	1215	1.2% (1.1 to 1.2)	1.2%
Referred at any point	12,692	16.6% (16.4 to 16.9)	16.4%	12,292	5.5% (5.4 to 5.6)	5.4%	2860	2.8% (2.7 to 2.9)	2.7%
CIN2 + ^a^	4883	6.3% (6.2 to 6.5)	6.1%	3810	1.7% (1.6 to 1.8)	1.5%	494	0.5% (0.4 to 0.5)	0.4%
CIN3 + ^a^	2903	3.8% (3.7 to 3.9)	3.6%	2344	1.0% (1.0 to 1.1)	0.9%	276	0.3% (0.2 to 0.3)	0.3%

*Abbreviations*. *CIN* Cervical intraepithelial neoplasia, *HPV* High-risk human papillomavirus

^a^CIN2 + includes CIN2, CIN3 and cervical cancer. CIN3 + includes CIN3 and cervical cancer

For the remaining six population characteristics indicators, the 95% CIs for the differences in means between the pilot and non-pilot areas all included zero and appeared small compared with the ranges observed over the entire country (Table 1).

The point estimates for differences in weighted means comparing HPV with LBC pilot areas were slightly larger than were those comparing pilot with non-pilot areas, but still relatively small and with greater uncertainty due to being based on fewer CCGs (Table 1). The direction of the differences suggested that HPV areas were less deprived than LBC areas, had fewer smokers, and a higher cervical screening coverage. However, the combined HPV and LBC areas for the two laboratory sites that were excluded from this comparison (consisting of 26% of the pilot’s population base) had a somewhat more favourable profile than did the included areas, with the weighted average IMD of 20.8 vs. 26.2, respectively, and the screening coverage of 74.7% vs. 71.9%, respectively. Hence, our inability to include in this comparison the catchment areas from the entire pilot may have affected the observed differences between HPV and LBC areas. Nevertheless, the individual-level data from the pilot reported previously support the hypothesis that women attending screening in HPV areas had less deprived backgrounds than women attending screening in LBC areas [6].

The analyses repeated using the 2013–2016 definition did not change our conclusions (Supplementary Information Table S4).

## Discussion

The degree to which data collected within an implementation pilot study will generalise to patient populations from non-pilot areas is not often directly addressed. We proposed and demonstrated the application of one method to help study this using publicly available area-level statistics. Our analysis identified some differences in population characteristics between the English HPV pilot and non-pilot areas, notably related to IMD. Overall, since process indicators in the pilot did not vary greatly by IMD, standardisation to account for differences between the pilot and wider population revealed only very small observed changes. This lends some support to extrapolation of the screening process indicators observed in the pilot across the rest of England.

Large differences in prevalence-round process indicators for HPV testing compared to LBC have been previously reported from the English pilot, for example a tripled test positivity with HPV testing, 80% higher colposcopy referral rate, and 50% higher CIN2 + detection [6]. As women in this implementation pilot were not individually randomised, these estimates were adjusted for age and IMD. Such an adjustment is consistent with the findings from the present analysis which suggested that the populations in the pilot’s HPV areas were less deprived than those in LBC areas. In our analysis, we also found some differences between the HPV and LBC areas in the prevalence of smoking and the uptake of screening. It is, however, unclear whether these associations suggest important residual confounding by population characteristics. There is, first, uncertainty about the estimated sizes and directions of these differences. Second, it is likely that the effect of smoking and screening coverage would be to some extent addressed through the adjustment for IMD, which has also been suggested in the stratified analysis (Supplementary Information Table S5) [6, 20].

Considering generalisability is important for both randomised and non-randomised implementation pilots. Pilot studies are sometimes embedded in pragmatic trials to help enable more robust evaluation of new technologies in comparison to current practice [42]. These trials might be based on individual randomisation, or cluster or step-wedge designs. However, unless the choice of locations is at random from the population of all locations, such trials may also have issues regarding generalisability or transportability to other health care units, particularly for uses where the absolute values of the observed parameters such as process indicators is critical. Planning of capacity and other commissioning decisions involved in implementation of a new health care technology is an example of such use of data. Non-randomised pilot studies are often done because randomisation within an implementation pilot may not always be feasible nor desirable [43]. For example, the English NHS tends to be organised by catchment areas, in the sense that funding and certain other decisions are made locally, with patients usually referred to local health care providers. In both randomised and non-randomised pilot studies methods are needed to transport findings from the study population to the target population. We presented the application of one statistical approach to help extrapolate to non-pilot areas. We did so by reweighing (standardising) the observed pilot data to better match the population served by the screening programme. In our analysis of a specific case, there was minimal impact on national capacity planning.

While reweighing itself is a relatively simple statistical procedure, the available population statistics applied post-hoc do not always provide the information with the required level of granularity. This was also seen in our analysis; we described the necessary adjustments and limitations of the data in detail in Methods. Some individual-level sociodemographic data is sometimes recorded routinely in programme information systems, but these sources are also usually limited. Whenever it is likely that the data collected through a pilot would be used to steer implementation capacity decisions, therefore, there clearly is a need to try to consider and plan for measures to enhance generalisability already at the stage when a pilot is being designed.

A limitation of our methodology is that it uses ecological comparisons from population characteristics, aggregated at an area level. For women included in the pilot, where both individual-level IMD quintile and the CCG-level IMD quintile were available, a comparison of both revealed similar broad trends, but also wide variation within each region that was only given a single CCG-level number in our analysis (data not tabulated). Ethnicity is a population characteristic that is associated with screening outcomes independent of IMD [44], but was not assessed here due to difficulties applying the relatively simple analysis to an indicator with wide variation in missing values by CCG. Ideally, the external validity of the pilot data would be retrospectively determined by comparing to the official CSP process indicator statistics reported at the national level [45]. At present, however, this comparison is complicated by two developments, a growing proportion of vaccinated women in the CSP and the disruption caused by the COVID-19 pandemic in 2020–2021, which in England coincided with the first year of the national HPV testing roll-out [46].

## Conclusion

Our analytical framework using publicly available ecological data might be helpful when assessing the degree to which data from implementation pilots generalise to non-pilot areas. This analytical framework suggested that the English HPV screening pilot is a valuable dataset comparing HPV testing with LBC that is reasonably well matched to the English CSP in the characteristics considered. Women included in the pilot have been screened with HPV testing for about five years longer than those who first underwent HPV testing as part of the national roll-out. Hence, with continued registration of screening and diagnostic events among the included population the pilot dataset could continue to signal any issues that need to be addressed to develop the national CSP.

## Supplementary Information

**Additional file 1:** **Table S1.** CCGs included in analyses using 2013-2015 and 2013-2016 definitions of pilot CCGs. **Table S2.** Definitions of health and screening indicators from the Fingertips database used in the analysis. **Table S3.** Observed values for screening process indicators in the English HPV pilot, by age group and IMD quintile. **Table S4.** Comparison of population characteristics for pilot vs. non-pilot and pilot HPV vs. pilot LBC areas. Pilot areas were defined using the 2013-2016 definition. The comparison of HPV and LBC pilot areas based on four laboratory sites. **Table S5.** Comparison of pilot vs. non-pilot areas by IMD quintile and the prevalence of smoking. Pilot areas were defined using the 2013-2016 definition. **Figure S1.** Map of pilot site catchment areas in 2013-2016, including newly acquired areas following laboratory mergers. **Figure S2.** Distribution of values across England for indicators included in the study. Definition of pilot CCGs using the 2013-2015 definition.

## Data Availability

The data from the pilot study belong to the former Public Health England and the authors cannot provide access to the relevant datasets to third parties. Requests for data and pre-application advice should instead be made to Office for Data Release (ODR@phe.gov.uk).

## Abbreviations

CCG: Clinical commissioning group
CI: Confidence interval
CIN: Cervical intraepithelial neoplasia
CSP: Cervical Screening Programme
GP: General practice
HPV: Human papillomavirus
IMD: Index of multiple deprivation
LBC: Liquid-based cytology
ODR: Office for Data Release
ONS: Office for National Statistics
STI: Sexually transmitted infection
