# Supplementary material for: Supporting the implementation of new healthcare technologies by investigating generalisability of pilot studies using area-level statistics
Source: BMC Health Serv Res. 2022 Nov 24;22:1412. doi: 10.1186/s12913-022-08735-3 (PMC9694587; doi:10.1186/s12913-022-08735-3)

Cancer Prevention Group

School of Cancer & Pharmaceutical Sciences

Faculty of Life Sciences & Medicine

King’s College London

London, 23 April 2022

**INVESTIGATING GENERALISABILITY OF IMPLEMENTATION PILOT STUDIES USING AREA-LEVEL STATISTICS:**

**A CASE STUDY OF THE ENGLISH HPV PILOT**

**SUPPLEMENTARY INFORMATION**

James A Doorbar, Christopher S Mathews, Karin Denton, Matejka Rebolj,

Adam R Brentnall, on behalf of the HPV Pilot Steering Group

**Summary**

This file contains additional information on the data used in the study; additional breakdowns of the data used in the study; and additional maps describing the distribution of health and screening indicator values across England.

Table S1. CCGs included in analyses using 2013-2015 and 2013-2016 definitions of pilot CCGs.

|  | **Pilot site** | **Primary screening test** |
| --- | --- | --- |
| **Pilot CCGs in 2013-2015** |  |  |
| NHS Barnsley CCG | Sheffield | LBC |
| NHS Bassetlaw CCG | Sheffield | LBC |
| NHS Blackburn with Darwen CCG | Manchester | LBC |
| NHS Blackpool CCG | Manchester | HPV |
| NHS Brent CCG | Northwick Park | LBC |
| NHS Bristol CCG | Bristol | Both |
| NHS Chorley and South Ribble CCG | Manchester | LBC |
| NHS Doncaster CCG | Sheffield | LBC |
| NHS Ealing CCG | Northwick Park | LBC |
| NHS East Lancashire CCG | Manchester | LBC |
| NHS Fylde and Wyre CCG | Manchester | HPV |
| NHS Great Yarmouth and Waveney CCG | Norwich | LBC |
| NHS Greater Preston CCG | Manchester | LBC |
| NHS Harrow CCG | Northwick Park | LBC |
| NHS Hillingdon CCG | Northwick Park | HPV |
| NHS Liverpool CCG | Liverpool | LBC |
| NHS Morecambe Bay CCG | Manchester | HPV |
| NHS North Cumbria CCG | Manchester | HPV |
| NHS North Norfolk CCG | Norwich | Both |
| NHS North Somerset CCG | Bristol | Both |
| NHS Norwich CCG | Norwich | Both |
| NHS Rotherham CCG | Sheffield | LBC |
| NHS Sheffield CCG | Sheffield | HPV |
| NHS South Gloucestershire CCG | Bristol | Both |
| NHS South Norfolk CCG | Norwich | Both |
| NHS South Sefton CCG | Liverpool | HPV |
| NHS Southport and Formby CCG | Liverpool | HPV |
| NHS West Lancashire CCG | Manchester | LBC |
| NHS West Norfolk CCG | Norwich | LBC |
| **Additional pilot CCGs in 2013-2016** |  |  |
| NHS Bolton CCG | Manchester | LBC |
| NHS Bury CCG | Manchester | LBC |
| NHS Heywood, Middleton and Rochdale CCG | Manchester | LBC |
| NHS Manchester CCG | Manchester | LBC |
| NHS Oldham CCG | Manchester | LBC |
| NHS Salford CCG | Manchester | LBC |
| NHS Stockport CCG | Manchester | LBC |
| NHS Tameside and Glossop CCG | Manchester | LBC |
| NHS Trafford CCG | Manchester | LBC |
| NHS Wigan Borough CCG | Manchester | LBC |

Abbreviations. HPV: high-risk human papillomavirus testing. LBC: liquid-based cytology. Both: both LBC and HPV testing were used in the CCG and were allocated by GP surgery.

Table S2. Definitions of health and screening indicators from the Fingertips database used in the analysis.

Source: Public Health England: Public Health Profiles 2021. URL: <https://fingertips.phe.org.uk>. Last accessed: 18 February 2021.

| **Indicator (measurement year)** | **Full name** | **IndicatorID** | **Source of data** | **Definitions** | **Area type for available data** |
| --- | --- | --- | --- | --- | --- |
| **Cervical screening coverage**  **(2014/15)** | Females, 25-64, attending cervical screening within target period | 91341 | NHAIS via the Open Exeter System, collected by the NHS Cancer Screening Programme | Num: number of women registered at the practice screened adequately in the previous 42 months (if aged 24-49) or 66 months (if aged 50-64). Denom: number of eligible women on last day of review period (after accounting for the exception rate for women who have had a hysterectomy involving the complete removal of the cervix). | CCG |
| **IMD score**  **(2015)** | Index of multiple deprivation score (IMD 2015) | 91872 | Department of Communities and Local Government | Score based on 38 separate indicators, see: <https://www.gov.uk/government/publications/english-indices-of-deprivation-2015-technical-report> | CCG |
| **Proportion smokers**  **(2014/15)** | QOF based smoking prevalence estimate | 91280 | QOF | Num: number of patients 15+ who are recorded as current smokers. Denom: estimated number of patients 15+. The proportion of patients without a recorded smoking status varies by practice from 40% to less than 1%. | CCG |
| **HPV vaccination coverage, % (2018/19)** | Population vaccination coverage - HPV vaccination coverage for two doses (females 13-14 years old) | 92896 | Local authority and area teams | All girls aged 13-14 years who have received the second (completing) dose of the HPV vaccine within each reporting area (local authority - LA) as a percentage of all girls aged 13-14 years within each area. | County |
| **Cervical cancer incidence (2011/13)** | Cervical cancer registrations rate / 100,000 | 90738 | The Information Centre for Health & Social Care (HSCIC) | Directly age-standardised rate of cervical cancer registrations per 100,000 female all age population. Num: Incidence of cervical cancer based on ICD10 code C53 in three combined calendar years. ICD10 information available at <http://apps.who.int/classifications/icd10/browse/2010/en#/II> Denom: three year combined mid-year estimates of the female resident population. | County |
| **New STI diagnoses rate/ 100,000 (2019)** | All new STI diagnosis rate / 100,000 | 91523 | Public Health England. Reported via the GUMCAD STI Surveillance System, the mandatory STI surveillance system for commissioned sexual health services in England. | All new STI diagnoses among people accessing specialist and non-specialist sexual health services in England. Data represent STI diagnoses among people who are resident in England. Data exclude people accessing services located in England who are resident in Wales, Scotland, Northern Ireland or abroad. Data are presented by area of patient residence, and are expressed as a rate per 100,000 population. | County |
| **Satisfaction with GP (2015/16)** | Percentage of people who said they had good experience when making a GP appointment | 92795 | GP Patient Survey (GPPS) | This indicator measures the weighted percentage of people who report their experience of  making a GP appointment as ‘fairly good’ or ‘very good’. Num: weighted number of people reporting a ‘fairly good’ or ‘very good’ experience of their GP surgery when answering the question 'Overall, how would you describe your experience of your GP surgery?'. Denom: total weighted number of people who answered the GP surgery experience question 'Overall, how would you describe your experience of your GP surgery?' | County |

Abbreviations. Denom=denominator. GP=general practice. IMD=index of multiple deprivation. NHAIS=National Health Application and Infrastructure Services. NHS=National Health Service. Num=numerator. QOF=Quality of Outcomes Framework. STI: sexually transmitted infections.

Table S3. Observed values for screening process indicators in the English HPV pilot, by age group and IMD quintile.

| **IMD quintile ↓** | **HPV positive** | | | **Baseline colposcopy referral** | | | **Any colposcopy referral** | | | **CIN2+** | | | **CIN3+** | | |
| --- | --- | --- | --- | --- | --- | --- | --- | --- | --- | --- | --- | --- | --- | --- | --- |
| **Age group →** | **24-29** | **30-49** | **50-64** | **24-29** | **30-49** | **50-64** | **24-29** | **30-49** | **50-64** | **24-29** | **30-49** | **50-64** | **24-29** | **30-49** | **50-64** |
| 1 | 25% | 8% | 6% | 12% | 3% | 1% | 15% | 5% | 2% | 5% | 1% | <1% | 3% | <1% | <1% |
| 2 | 25% | 9% | 5% | 10% | 3% | 1% | 15% | 5% | 3% | 5% | 1% | <1% | 3% | 1% | <1% |
| 3 | 27% | 10% | 5% | 11% | 3% | 1% | 17% | 5% | 2% | 7% | 2% | 1% | 4% | 1% | <1% |
| 4 | 28% | 12% | 7% | 9% | 3% | 1% | 16% | 6% | 4% | 5% | 1% | <1% | 4% | 1% | <1% |
| 5 | 28% | 11% | 6% | 13% | 4% | 2% | 18% | 6% | 3% | 7% | 2% | 1% | 4% | 1% | <1% |

Abbreviations. CIN: cervical intraepithelial neoplasia.

Note: The denominator for proportions in the table were women screened with HPV testing. IMD quintile defined as describe in Methods. IMD quintile 1: least deprived. IMD quintile 5: most deprived.

Table S4. Comparison of population characteristics for pilot vs. non-pilot and pilot HPV vs. pilot LBC areas. Pilot areas were defined using the 2013-2016 definition. The comparison of HPV and LBC pilot areas based on four laboratory sites.

| **Indicator** | **Pilot**  **(weighted mean, SD)** | **Non-pilot (weighted mean, SD)** | **Difference pilot vs. non-pilot**  **(95% CI)** | **HPV testing**  **(weighted mean, SD)** | **LBC (weighted mean, SD)** | **Difference HPV vs. LBC**  **(95% CI)** |
| --- | --- | --- | --- | --- | --- | --- |
| IMD score | 25.9 (7.6) | 20.9 (7.9) | 5.0 (1.9 to 8.0) | 24.2 (6.3) | 27.9 (7.6) | -3.7 (-9.0 to 1.8) |
| Smoking prevalence | 19.6 (2.8) | 18.2 (2.6) | 1.5 (0.4 to 2.5) | 18.4 (2.3) | 20.2 (2.8) | -1.8 (-3.4 to 0.4) |
| Cervical screening coverage | 72.7 (4.1) | 73.9 (3.9) | -1.2 (-2.8 to 0.3) | 73.5 (3.4) | 71.8 (4.4) | 1.7 (-2.0 to 4.5) |
| HPV vaccination | 83.5 (7.9) | 83.7 (6.7) | -0.2 (-3.9 to 3.1) |  |  |  |
| Incidence of STI per 100,000 | 789.3 (316.3) | 802.7 (526.6) | -13.5 (-173.6 to 151.0) |  |  |  |
| Incidence of cervical cancer per 100,000 | 10.5 (2.6) | 9.8 (2.1) | 0.7 (-0.3 to 1.8) |  |  |  |
| Satisfaction with GP surgery | 72.9 (3.3) | 73.4 (4.9) | -0.5 (-2.2 to 1.0) |  |  |  |

Abbreviations. GP: general practice/practitioner. HPV: human papillomavirus. IMD: Index of Multiple Deprivation. STI: sexually transmitted infections.

Interpretation

This analysis shows similar patterns as the analysis where CCG areas were defined following the 2013-2015 definition. Pilot area showed greater levels of deprivation. There was less evidence of a difference between pilot and non-pilot areas by smoking prevalence, but non-pilot areas had lower levels. To investigate the extent to which deprivation might explain this, we conducted a stratified analysis, comparing smoking prevalence by IMD quintile (Table S5). This suggested that IMD-standardised estimates would account for much of any potential differences in smoking. That is, IMD and smoking standardised estimates would be very similar to those standardised for IMD only.

Table S5. Comparison of pilot vs. non-pilot areas by IMD quintile and the prevalence of smoking. Pilot areas were defined using the 2013-2016 definition.

| IMD quintile | **Pilot**  **(weighted mean, SD)** | **Non-pilot (weighted mean, SD)** | **Difference pilot vs. non-pilot**  **(95% CI)** |
| --- | --- | --- | --- |
| 1 (least deprived) | 14.5 (1.4) | 15.3 (1.2) | -0.8 (-2.5 to 0.9) |
| 2 | 17.1 (0.8) | 17.1 (0.9) | 0.0 (-0.6 to 0.7) |
| 3 | 18.3 (1.2) | 18.2 (1.5) | 0.1 (-0.8 to 1.1) |
| 4 | 19.7 (2.2) | 20.0 (1.7) | -0.4 (-1.9 to 1.2) |
| 5 (most deprived) | 22.6 (1.3) | 21.2 (1.7) | 1.4 (0.5 to 2.3) |

Figure S1. Map of pilot site catchment areas in 2013-2016, including newly acquired areas following laboratory mergers.

The figure was generated by authors using the QGIS software with information from the sources explained in the Methods.


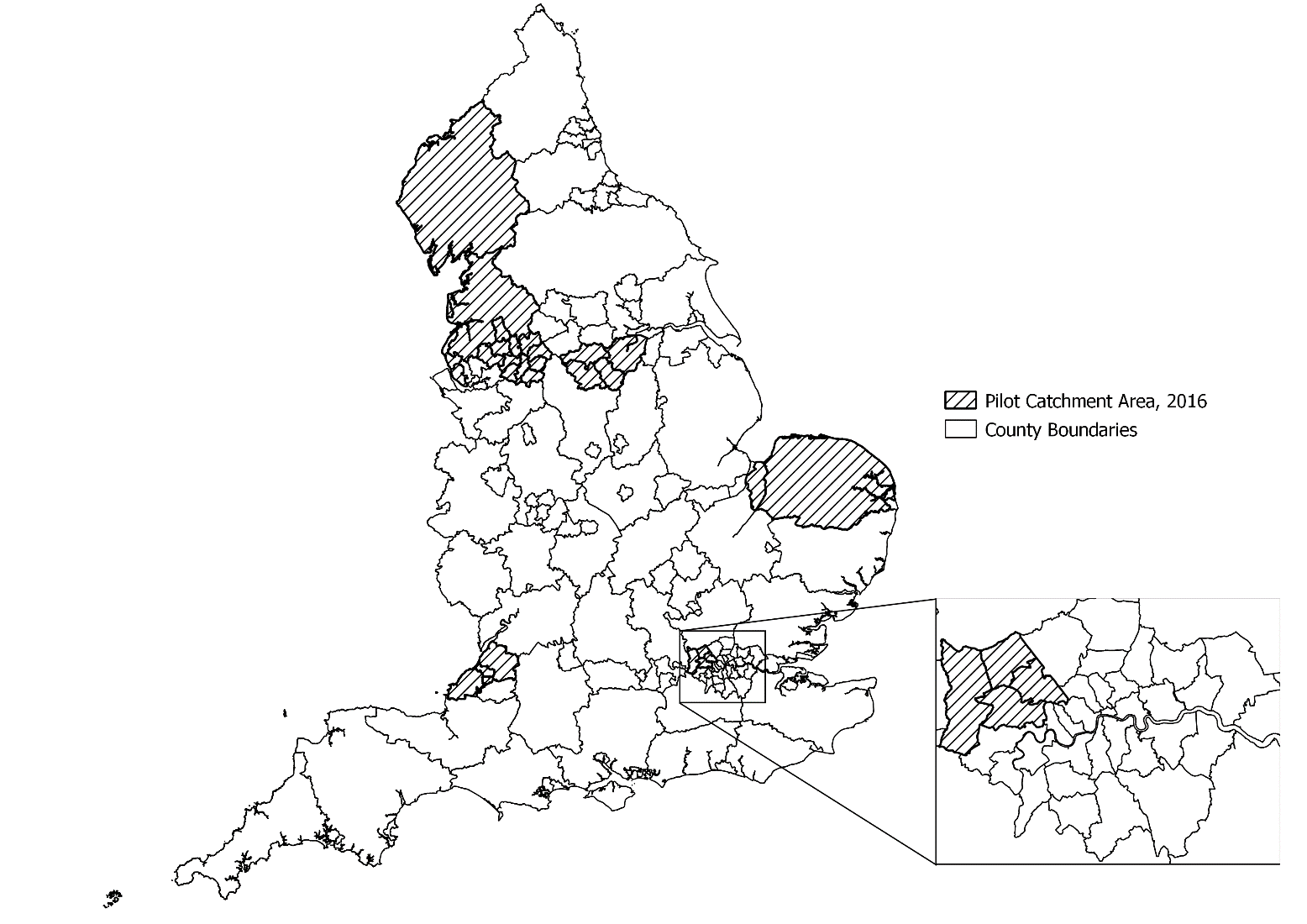


Figure S2. Distribution of values across England for indicators included in the study. Definition of pilot CCGs using the 2013-2015 definition. Panel A: smoking prevalence. Panel B: age-appropriate cervical screening coverage. Panel C: HPV vaccination coverage. Panel D: incidence of sexually transmitted infections. Panel E: incidence of cervical cancer. Panel F: satisfaction with GP surgery.

The figures were generated by authors using the QGIS software with information from the sources explained in the Methods.

A


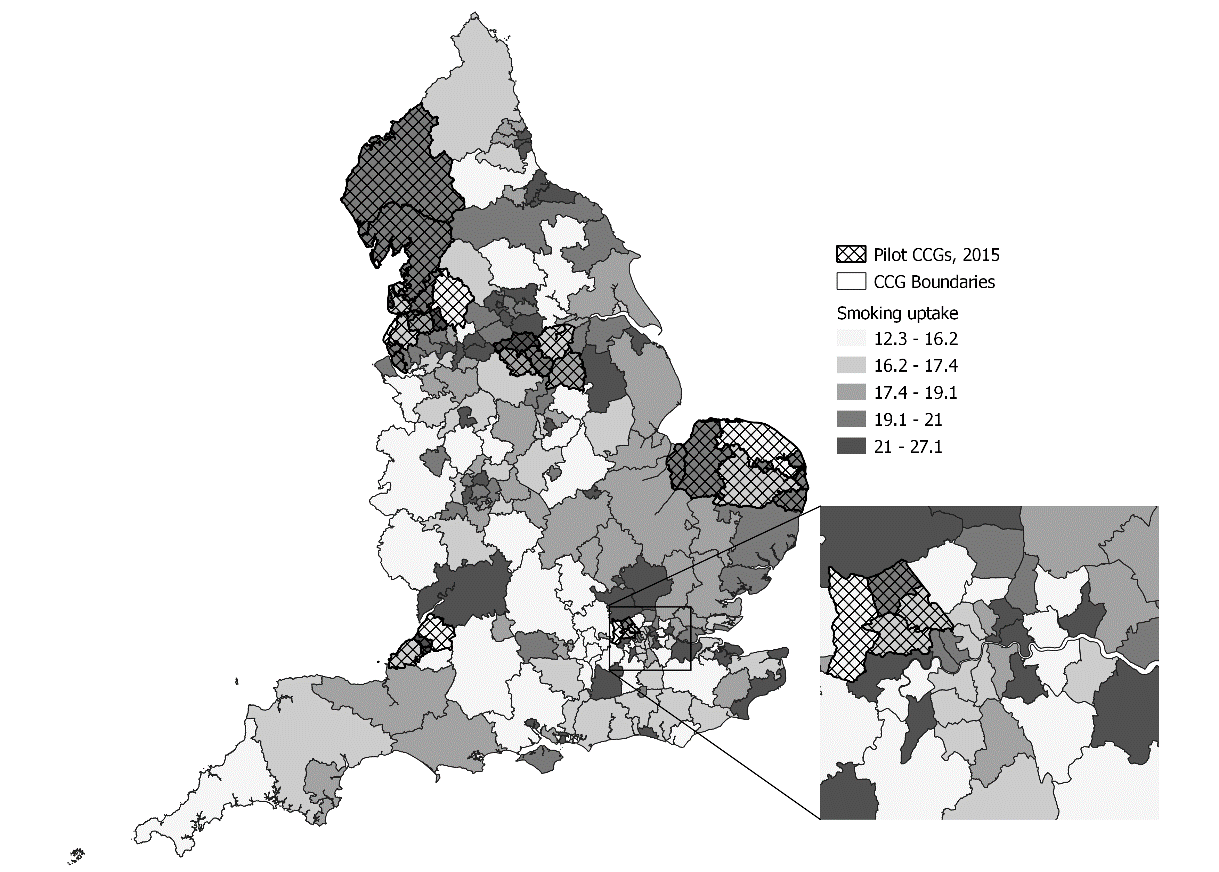


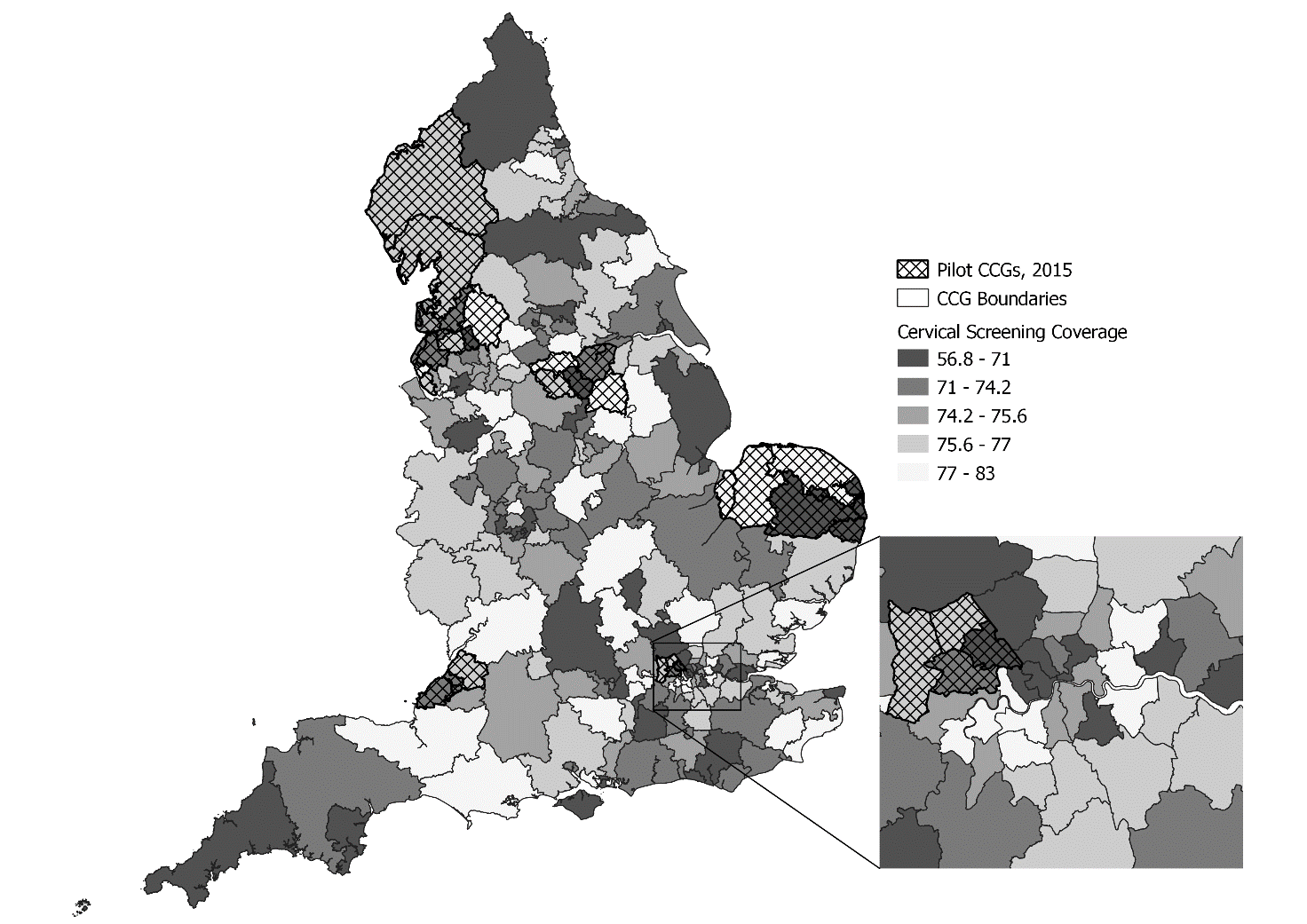
B


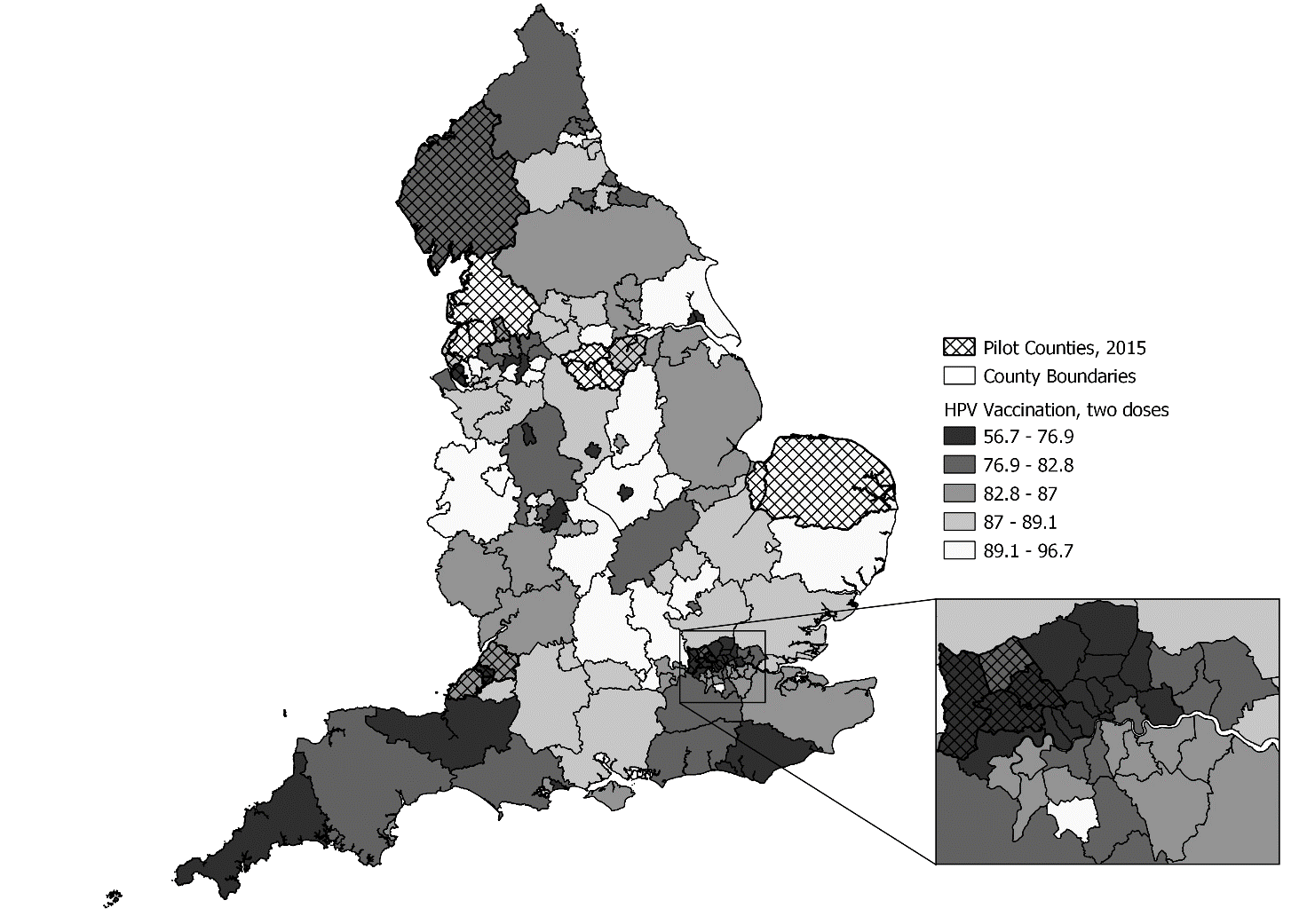
C

D


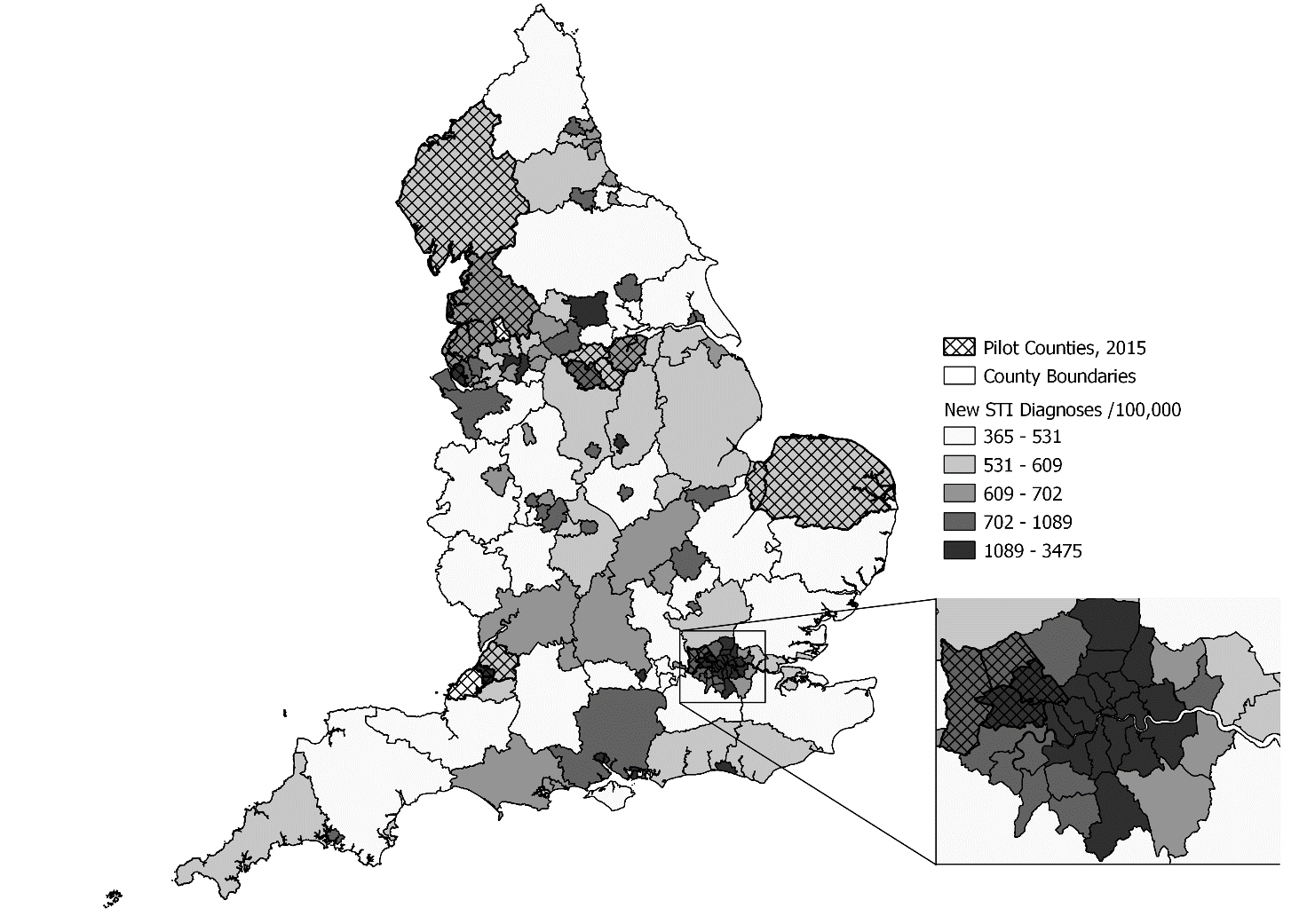


E


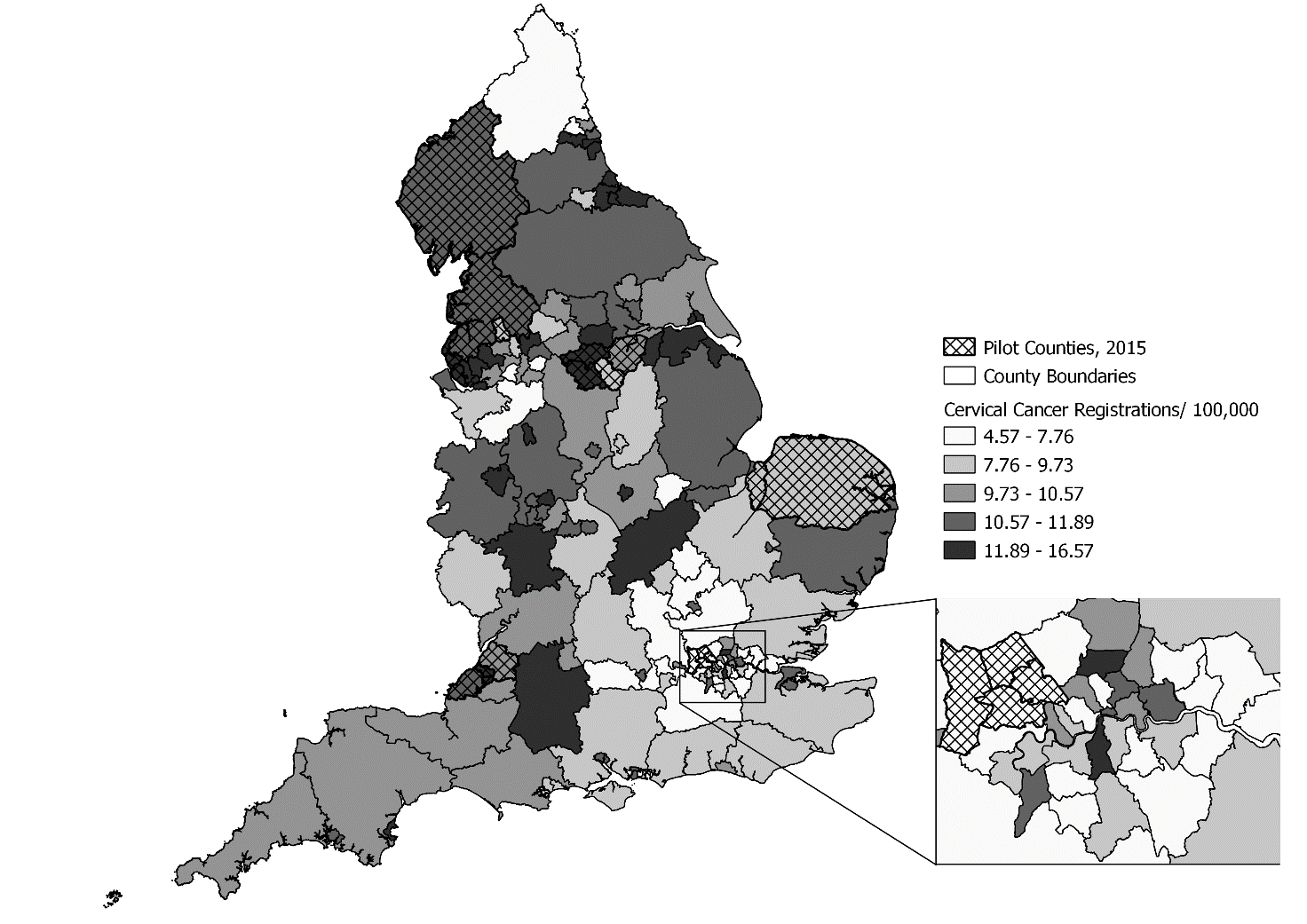


F


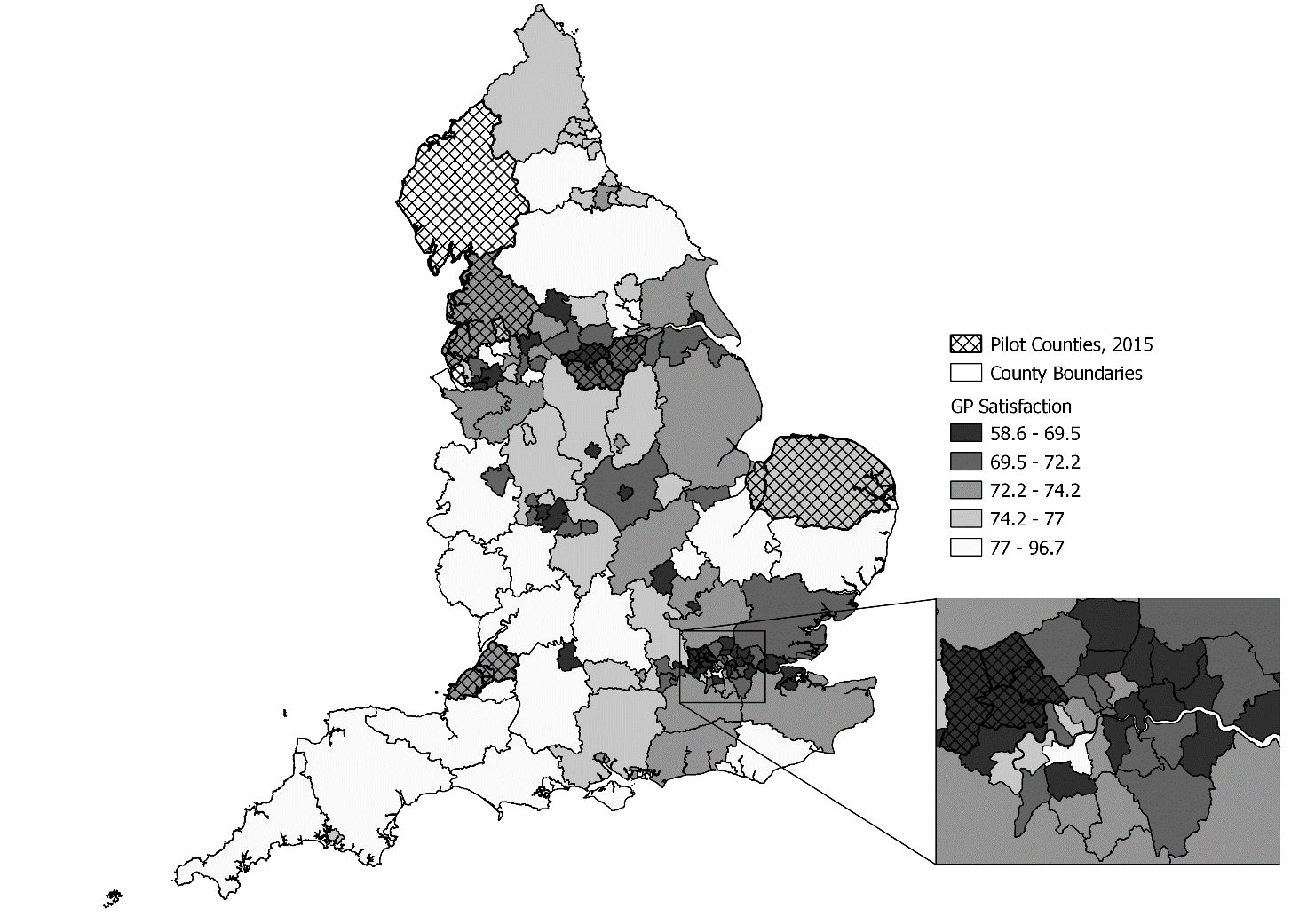

Supplement: Supplementary file 1 — Additional file 1: Table S1. CCGs included in analyses using 2013-2015 and 2013-2016 definitions of pilot CCGs. Table S2. Definitions of health and screening indicators from the Fingertips database used in the analysis. Table S3. Observed values for screening process indicators in the English HPV pilot, by age group and IMD quintile. Table S4. Comparison of population characteristics for pilot vs. non-pilot and pilot HPV vs. pilot LBC areas. Pilot areas were defined using the 2013-2016 definition. The comparison of HPV and LBC pilot areas based on four laboratory sites. Table S5. Comparison of pilot vs. non-pilot areas by IMD quintile and the prevalence of smoking. Pilot areas were defined using the 2013-2016 definition. Figure S1. Map of pilot site catchment areas in 2013-2016, including newly acquired areas following laboratory mergers. Figure S2. Distribution of values across England for indicators included in the study. Definition of pilot CCGs using the 2013-2015 definition. [file 12913_2022_8735_MOESM1_ESM.docx]
